# Profile of Childhood Poisoning and Its Outcomes in the United States: A One-Year Nationwide Study of Emergency and Inpatient Admissions

**DOI:** 10.7759/cureus.37452

**Published:** 2023-04-11

**Authors:** Fidelis Uwumiro, Victory Okpujie, Oluwatobi A Olaomi, Olawale Abesin, Festa C Madu, Nsikan N Akpabio, Michael I Otu, Michael M Bojerenu, Folajimi J Atunde, Ayodeji Ilelaboye

**Affiliations:** 1 Medicine and Surgery, Our Lady of Apostles Hospital, Akwanga, NGA; 2 Internal Medicine, Central Hospital, Benin City, NGA; 3 Medicine and Surgery, University of Ibadan, Ibadan, NGA; 4 Internal Medicine, Royal Cornwall Hospital, NHS (National Health Service) Trust, Truro, GBR; 5 Internal Medicine, Nnamdi Azikiwe University, Awka, NGA; 6 Medicine and Surgery, Bingham University Teaching Hospital, Jos, NGA; 7 Medicine and Surgery, University of Calabar, Calabar, NGA; 8 Internal Medicine, St. Barnabas Hospital, SBH (St. Barnabas Hospital) Heath System, New York City, USA; 9 Neurology, NES Healthcare, Aylesbury, GBR; 10 Medicine and Surgery, Ladoke Akintola University of Technology, Ogbomosho, NGA

**Keywords:** household poisoning, nationwide emergency department sample, nationwide inpatient sample, drug overdose, chilhood poisoning

## Abstract

Childhood poisoning is a prevalent and significant public health issue, with a higher incidence among children under the age of five due to their natural inquisitiveness and impulsive behavior. In order to gain a better understanding of the burden and outcomes of acute poisoning in children, this study utilized data from two comprehensive databases: the 2018 Nationwide Emergency Department Sample and the National (Nationwide) Inpatient Sample. A total of 257,312 hospital visits were analyzed, with 85.5% being emergency department visits and 14.5% being inpatient admissions. Drug overdose emerged as the most commonly known cause of poisoning in both emergency and inpatient settings. While alcohol poisoning was the predominantly known cause of non-pharmaceutical poisoning in the inpatient setting, household soaps and detergents were more common in the emergency setting. Among the identified pharmaceutical agents, non-opioid analgesics and antibiotics were the most frequently implicated. However, a significant proportion of the poisoning cases were caused by unidentified substances (26.8% in the pharmaceutical group and 72.2% in the non-pharmaceutical group). There were 211 deaths in total and further analysis revealed that patients with higher Charlson indices and hospital stays exceeding seven days were associated with increased likelihood of mortality. Additionally, admission to teaching hospitals or hospitals located in the western region of the country was linked to an increased likelihood of an extended hospital stay.

## Introduction

Children are vulnerable to unintentional poisoning in and around their homes and may be inclined to explore a variety of household items due to their natural curiosity. This behavior has resulted in millions of calls to poison control centers annually, and thousands of children being admitted to emergency departments after inadvertently consuming household products, medicines, or pesticides. However, many of these incidents are preventable with proper precautions [1]. According to the 2022 United States (US) Consumer Product Safety Commission's (CPSC) annual report on pediatric poisoning fatalities and injuries, unintentional poisoning from household consumer products results in an average of 31 deaths of children under five years of age each year. This figure is reported by the CPSC to represent an 80% decrease from the 216 fatalities reported in 1972.

Age is a critical factor in both the likelihood and severity of poisoning [2,3]. The toxicity of many substances tends to increase with higher doses in relation to body mass, and the body's enzyme systems responsible for eliminating certain toxins tend to become more effective as the child ages. Given many cases of poisoning are unintentional, children are particularly susceptible due to their natural curiosity and tendency to put unknown substances into their mouths.

The primary objective of this study is to provide a comprehensive overview of childhood poisoning and its related outcomes in the US, using nationally representative data. Specifically, the study aims to achieve five objectives: firstly, to depict the profile of childhood poisoning based on patient demographics; secondly, to examine the distribution of childhood poisoning by etiology; thirdly, to assess the magnitude of emergency department (ED) visits and inpatient admissions for poisoning in children; fourthly, to investigate the mortality rate and mean length of hospital stay (LOS) for emergency and inpatient admissions related to poisoning in children; and finally, to identify any autonomous predictors of in-hospital mortality and prolonged hospital admission.

## Materials and methods

Data source

In this study, we utilized two comprehensive databases, namely the 2018 Nationwide Emergency Department Sample (NEDS) and the National (Nationwide) Inpatient Sample (NIS). The NIS encompasses data from 47 states and the District of Columbia, effectively representing over 97% of the US populace and almost 96% of discharges from community hospitals. This database provides a wide range of clinical and resource utilization information that is typically included in discharge abstracts [4]. On the other hand, the NEDS is the largest all-payer ED database publicly available in the US, containing information from 35.8 million ED visits at 990 sampled hospital-owned EDs. We used weights to calculate national estimates, which represented about 143 million ED visits in the US in 2018. To ensure a representative sample of all US hospital-owned EDs, the NEDS stratifies important hospital characteristics such as geographic region, trauma center designation, urban-rural location, teaching status, and hospital ownership [5].

Ethical consideration

The US Agency for Healthcare Research and Quality (AHRQ) oversees the NIS and NEDS databases, which are part of the Healthcare Cost and Utilization Project (HCUP). These databases comply with the Health Insurance Portability and Accountability Act of 1996 (HIPAA) and exclude 16 direct patient and hospital identifiers. As limited data sets, NIS and NEDS do not require review by an institutional review board [6,7].

Inclusion criteria and study variables

All pediatric (age 0-18 years) admissions for poisoning were identified using the International Classification of Diseases, 10th Revision, Clinical Modification/Procedure Coding System (ICD-10-CM/PCS) and sub-classified into drug overdose (pharmaceutical) and non-drug-related (non-pharmaceutical) causes. Age categories were defined as 0-5, 6-10, 11-15, and 16-18 years. The study variables included various patient and hospital demographics such as age, gender, race, day of admission, hospital region, and hospital teaching status. The Charlson Comorbidity Index (CCI) was used to assess the burden of common pediatric comorbidities. Prolonged LOS was defined as LOS in the top decile for the total study population (> 7 days for inpatient admission and > 3 days for ED visits).

Outcome measures

The population statistics comprised the incidence of poisoning categorized by the type of poisoning, type of admission, patient’s age group, hospital region, and hospital teaching status. The primary outcome measure was the mortality of patients during hospitalization or in the ED. The secondary outcome measure was the average LOS.

Statistical analyses

Statistical analysis was performed using Stata® Version 17 (Released 2021; StataCorp LLC, College Station, Texas). Unadjusted odds ratios (ORs) were computed for the primary and secondary outcomes by means of univariate logistic regression analyses, incorporating all variables and co-morbidities. Subsequently, variables with p-values less than 0.1 (female sex, race, CCI, weekend admission, hospital region, hospital bed size, hospital teaching status, and prolonged LOS) were chosen for the ensuing multivariate logistic regression model. Proportions were compared using Fisher's exact test, while Student's t-test was used for continuous variables. The significance level for the multivariate analysis was set at p-values less than 0.05. Categorical and continuous variables were reported as proportions or mean with standard deviation, whereas regression outcomes were reported as adjusted ORs (AORs) or β coefficients with 95% confidence intervals (CIs).

## Results

Sociodemographic characteristics of the study population

Table 1 presents a comprehensive overview of the sociodemographic characteristics of the study population. Throughout the study period, a total of 257,312 hospital visits resulting from poisoning in children were documented. Notably, a larger proportion of cases were reported in the ED (85.5%) than in inpatient care (14.5%). In both settings, drug overdosing accounted for the vast majority of cases of poisoning in children (81.6% vs. 92.9%, respectively). The data further revealed that a substantial number of children admitted to the ED were within the age range of 0-5 years (45.6%). In contrast, the age range for inpatient admissions was primarily between 11-18 years (66.7%).

**Table 1:** Sociodemographic characteristics of emergency visits and inpatient admissions for acute poisoning USD, United States dollar; CCI, Charlson comorbidity index; ED, emergency department

Variable	Emergency visits (n=220,087; 85.5%)	Inpatient admissions (n=37,225; 14.5%)
Pharmaceutical poisoning, %	81.6	92.9
Non-pharmaceutical poisoning, %	18.4	7.1
Sex, %		
Female	58.2	65.1
Male	41.8	34.9
Mean age (Years)	8.5 ± 0.7	11.5 ± 0.1
Age categories, %		
0-5 years	45.6	25.5
6-10 years	8.4	7.8
11-15 years	19.7	30.0
15-18 years	23.3	36.7
Race/ethnicity, %		
White	76.3	56.8
Black	13.4	14.9
Hispanic	5.9	18.3
Asian or Pacific Islander	1.3	2.7
Native American	0.2	1.7
Others	5.2	5.5
Weekend admission, %	27.5	25.0
CCI score , %		
0	71.6	83.9
1	16.2	13.0
2	8.5	2.0
≥3	3.7	1.1
Insurance type, %		
Medicaid	0.2	0.3
Medicare	58.5	53.8
Private	35.7	42.1
Uninsured	5.6	3.8
Median annual income in patient’s zip code, %		
1-43,999 USD	32.1	28.1
44,000-55,999 USD	28.2	26.6
56,000-73,999 USD	22.1	25.1
≥ 74,000 USD	17.5	20.2
Comorbidities, %		
Pediatric heart failure	0.04	0.3
Chronic renal disease	0.06	0.4
Diabetes	1.7	1.6
Asthma	9.2	4.6
Cancer	2.3	1.4
Liver disease	0.11	1.2
Crohn’s disease	0.04	0.01
Hospital characteristics, %		
Hospital region		
Northeast	13.4	14.1
Midwest	26.5	27.1
South	40.7	38.9
West	19.4	19.9
Rural	20.0	3.4
Urban non-teaching	64.7	6.4
Teaching hospital	15.3	90.2

Emergency department visits

According to the data, there were 220,087 ED visits resulting from poisoning in children. The causative agents of poisoning were categorized into pharmaceutical causes or drug overdose (81.6%) and non-pharmaceutical causes (18.4%), (Table 2). Non-pharmaceutical poisoning was most commonly caused by food poisoning (13.7%), ingestion of household soaps and detergents (11.6%), inhalation of toxic gases (7.8%), alcohol (5.8%), and organophosphates poisoning (4.7%). In 42.1% of cases, the responsible agent of poisoning was unknown to patients or their family members. Among cases of drug overdose, the most frequently identified types were non-opioid analgesics (21.5%), antibiotics (18.9%), tricyclic antidepressants (9.8%), drugs prescribed for hematological disorders (9.0%), and antiepileptics (8.4%). However, in 15.5% of drug-related poisoning cases, the responsible drug could not be identified by patients or their family members.

**Table 2:** Distribution of poisoning cases by type of hospital visit †Drugs prescribed for diseases of the ear, nose, and throat ‡Includes lead, iron, arsenic, cadmium, and mercury ɸIncludes carbon monoxide and other toxic gases ѱFurniture stripper, turpentine, charcoal lighter fluid, dry-cleaning fluids, paint thinner, nail polish remover, degreasers, and lubricating oils

Variable	Emergency visit	Inpatient admission
Pharmaceutical Poisoning, n (%)	179,591 (81.6%)	34,582 (92.9%)
Non-opioid analgesics, %	21.5	3.3
Antibiotics, %	18.9	24.1
Other unidentified drugs, %	15.5	11.3
Tricyclic antidepressants, %	9.8	16.6
Drugs for hematological disorders, %	9.0	0.0
Antiepileptic drugs, %	8.4	10.5
Cardiovascular drugs, %	5.5	6.2
Butyrophenone, %	4.5	7.7
Psychostimulant, %	3.9	5.2
Anticholinergics, %	3.4	3.4
Musculoskeletal agents, %	3.1	3.0
Hormones, %	2.6	3.4
ENT drugs^†^, %	2.2	0.5
Opioid analgesics, %	1.9	3.3
Drugs acting on the gastrointestinal tract, %	1.3	1.1
Anti-infective drugs, %	0.3	0.3
Anesthetic agents, %	0.2	0.1
Phenothiazines, %	0.2	0.0
Non-pharmaceutical Poisoning, n (%)	40,496 (18.4%)	2,660 (7.1%)
Unidentified substances, %	42.1	30.1
Food poisoning, %	13.7	5.8
Household soaps, %	11.6	4.5
Gases^ɸ^, %	7.8	13.1
Alcohol, %	5.8	26.3
Organophosphates, %	4.7	1.1
Household solvents^ѱ^, %	4.1	2.6
Tobacco & nicotine, %	2.8	0.9
Heavy metals^‡^, %	0.8	10.0
Latex, %	0.3	0.2
Inorganic compounds, %	0.2	0.6
Seafood, %	0.2	0.2
Cyanide, %	0.04	0.4
Venoms, stings, and bites, %	0.01	0.0

Inpatient admissions

There were 37,225 hospitalizations due to poisoning in children. Most admissions (60.5%) had evidence of having received emergency care in the same hospital while 39.4% transferred from other acute care facilities. The causative agents of poisoning were divided into two main categories: pharmaceutical causes or drug overdose (n=34,582, 92.9%) and non-pharmaceutical causes (n=2,660, 7.1%), as shown in Table 2.

The leading types of non-pharmaceutical poisoning were alcohol (26.3%), toxic gases (13.1%), heavy metals (10%), food poisoning (5.8%), household soaps (4.5%), and household solvents (2.6%). In 800 (30.1%) cases, the causative agents remained unidentified by the patients or their family members. In the category of drug overdose, the major types identified included non-opioid analgesics (29%), antibiotics (24%), tricyclic antidepressants (16.6%), antiepileptics (10.5%), cardiovascular drugs (6.2%), psychostimulants (5.2%), and butyrophenones (7.7%). In 3,908 (11.3%) cases, the causative agents could not be identified by the patients or their family members.

Outcomes

Mortality

During the study year, the ED data recorded a total of 77 deaths, with a majority of them (44, 57.1%) being females. Of these deaths, 41 (53.2%) were attributed to drug overdose, while 36 (46.8%) were due to non-drug-related poisonings. Among patients admitted to inpatient care, there were 134 mortalities, which accounted for 0.36% of the admissions. Pharmaceutical poisoning or drug overdose was the cause of 90 of these inpatient deaths, while non-pharmaceutical poisoning caused the remaining 44 deaths. Most inpatient mortalities were observed in males (99.7%), aged 5-10 years (59%), of Asian or Pacific Islander race (52%), and had a CCI of 2 or less (80.3%). Additionally, 40% of the deceased were uninsured and were admitted to large hospitals in the northeast (41%). In-hospital mortality was independently associated with hospital admission lasting longer than seven days or a higher CCI (AOR: 6.54 and 1.48, respectively; p<0.001). In comparison, the female sex was found to independently predict lower mortality in the ED (AOR: 0.26; 95%CI: 0.08-0.80; p=0.02).

LOS

The average LOS for patients admitted to the ED due to poisoning was 3.8 ± 0.2 days. The majority of these patients were hospitalized for three days or less, accounting for 75.4% of the total admissions. For all patients admitted to inpatient care, the average LOS was 4.5 days, with a majority of the admissions (71.9%) also lasting three days or less. The distribution of hospital admissions that exceeded three days is presented in Table 3, categorized by age group.

**Table 3:** Distribution of prolonged hospital stay by age category and hospital region *Admission exceeding three days for emergency department visits or seven days in inpatient care

	Emergency visits	Inpatient admissions
Total prolonged hospital admission,^* ^n (%)	54,141 (24.6%)	10,460 (28.1%)
Age category, %		
0-5 years	50.8	26.0
6-10 years	8.5	10.8
11-15 years	18.7	35.8
15-18 years	21.8	41.1
Hospital region, %		
Northeast	14.2	20.4
Midwest	29.1	38.2
South	43.9	48.3
West	21.5	23.4

The results of the study indicate that for inpatient admissions, there is a significant association between weekend admissions and a reduced likelihood of an admission longer than three days (AOR: 0.75; 95%CI: 0.67-0.85; p<0.001). Furthermore, the analysis reveals that a higher CCI and admission to teaching hospitals or hospitals in the west are linked to an increased probability of an extended hospital stay (p<0.001). Notably, female sex, older age, higher median household income quartiles for the patient's ZIP (Zone Improvement Plan) code, and admission to non-teaching facilities are all associated with lower odds of prolonged admissions (p<0.001).

## Discussion

Despite notable advancements in the last 30 years, this study reveals that emergency and hospital admissions due to poisoning persist and continue to pose a significant risk of mortality to hundreds of children annually. Furthermore, the research demonstrates that there are discrepancies in the age distribution of children admitted to the ED compared to those who require inpatient care. Specifically, the majority of ED admissions involved children aged 0-5 years, whereas inpatient admissions were primarily for children aged 11-18 years. These findings suggest that while children under the age of five are more susceptible to presenting to the ED with acute poisoning [8], those aged 11 and above are more prone to experiencing severe poisoning that necessitates inpatient admission [9].

Since the 1970s, efforts have been made to reduce childhood poisoning and its associated mortality in the US. These efforts include establishing Poison Control Centers (PCCs) that provide information, advice, and emergency treatment recommendations and facilitate patient transfers for urgent care [10]. The CPSC mandated child-resistant packaging for certain household products, resulting in a reduction in accidental poisonings in young children [11]. Public education campaigns aimed at parents and caregivers have also been effective in raising awareness about the safe storage of household products, proper medication use, and identification of toxic substances [12]. The regulation of hazardous substances through legislation such as the Toxic Substances Control Act [13] and the Poison Prevention Packaging Act [14] has also been an important strategy. Also, the Centers for Disease Control and Prevention monitor and report cases of childhood poisoning through the National Poison Data System. In recent years, some manufacturers have made product design changes, such as developing single-dose packaging for medications, to reduce the risk of poisoning. Overall, the efforts undertaken to alleviate childhood poisoning and its associated mortality in the US over the last 30 years have yielded some favorable outcomes. Nevertheless, given the incidence of poisoning and related fatalities reported in the present study, further research is necessary to identify more effective countermeasures that can prevent childhood poisoning and ensure the health and welfare of minors, surpassing the current achievements. 

The index study has several limitations that need to be acknowledged. Firstly, the use of the NIS and NEDS is subject to coding errors and insufficient information on intensity of care is provided. Additionally, these databases solely rely on admissions, thereby precluding outpatient visits that may relate to minor incidents of poisoning, among other pertinent factors. Furthermore, it is worth noting that these database-based inquiries are limited to hospitalizations and not individual patients. Consequently, patients who are admitted multiple times are treated as separate cases. Secondly, this study did not explore the distinction between intentional (suicidal or homicidal) and unintentional poisoning in children. Nevertheless, the study provides valuable insights into the prevalence, causes, and outcomes of childhood poisoning. These findings underscore the critical importance of advancing superior prevention strategies and educating parents and caregivers on drug safety and household poisoning prevention.

## Conclusions

Despite recent improvements in outcomes for childhood poisoning, this study highlights the fact that it still represents a significant public health problem in the US. Every year, hundreds of thousands of children are at risk of mortality due to poisoning. While past prevention efforts have had some success in reducing the incidence and associated deaths from childhood poisoning, this study emphasizes the need for continued investment in prevention initiatives. Future research should prioritize identifying effective prevention measures, as well as examining other factors that contribute to poisoning, especially in older children who may be more likely to intentionally poison themselves. Evaluating the impact of current prevention efforts can help inform future policies and interventions. Additional value could be gained by exploring the influence of psychosocial factors on intentional poisoning in older children in future research.
